# A new gene order in the mitochondrial genome of the deep-sea diaphanous hatchet fish *Sternoptyx diaphana* Hermann, 1781 (Stomiiformes: Sternoptychidae)

**DOI:** 10.1080/23802359.2020.1790325

**Published:** 2020-07-15

**Authors:** Nair Vilas Arrondo, André Gomes-dos-Santos, Esther Román Marcote, Montse Pérez, Elsa Froufe, L. Filipe C. Castro

**Affiliations:** a AQUACOV, Instituto Español de Oceanografía, Centro Oceanográfico de Vigo, Vigo, Spain; b Faculty of Biology, UVIGO, PhD Program “Marine Science, Technology and Management” (Do*MAR), University of Vigo, Vigo, Spain; c CIIMAR/CIMAR – Interdisciplinary Centre of Marine and Environmental Research, University of Porto, Matosinhos, Portugal; d Department of Biology, Faculty of Sciences, University of Porto, Rua do Campo Alegre, Porto, Portugal; e BIOPESLE Español de Oceanografía, Centro Oceanográfico de Vigo, Vigo, Spain

**Keywords:** Stomiiformes, Sternoptychidae, mtDNA, deep-sea, Diaphanous hatchet fish

## Abstract

Species of the Sternoptychidae teleost family display an impressive morphology, including their extreme reduced size. Here, we report the first mitochondrial genome of the diaphanous hatchet fish *Sternoptyx diaphana*. By using short-read sequencing Illumina HiSeq, we generated two mitochondrial contigs which were later physically assembled by PCR. The mitochondrial genome of S. diaphana was 17,224 bp in length (excluding the control region) and is composed of 13 PCGs and 2 ribosomal RNA genes. Strikingly, we could not identify the tRNA-Phe and two copies of tRNA-Met were differently positioned. Additionally, the mitogenome displays a completely new gene rearrangement among vertebrates. We expect that the study presented here will pave the way for further molecular studies with this underrepresented group of illusive teleost fish.

Members of the marine teleost Sternoptychidae family (Stomiiformes) are characterized by their extremely small size (<100 mm). Moreover, they display a bright silver lateral pigmentation, large eyes, numerous lateral and ventral photophores, and a highly variable body morphology which is gender-specific (Nelson et al. 2016). The family consists of two subfamilies, the Maurolicinae and Sternoptychinae (Weitzman 1974), which include 70 species distributed over 10 genera. One of these species *Sternoptyx diaphana* Hermann 1781, is a very small (up to 45 mm) deep-sea ray-finned fish, with large eyes and short snout, and a laterally compressed body, with the mouth being nearly vertical.

A specimen of *S. diaphana* was captured in North Atlantic (48.0695 N; 47.0857 W) from the EU Groundfish Survey (Fletán Negro 3 L-2019) and stored in ethanol 96%. Due to its small dimension, the whole individual (except the digestive tract) was used for total genomic DNA extraction and subsequent whole-genome library preparation (350 bp Truseq DNA PCR-free Illumina kit) and sequencing (150 bp Paired-end on HiseqX150) done at Macrogen Inc., Korea. Nevertheless, other specimens of the same species simultaneously collected are stored in ethanol at Interdisciplinary Center of Marine and Environmental Research and the Instituto Español de Oceanografía, Centro Oceanográfico de Vigo with vouchers S.DIAPNA_1_3L19 and S.DIAPNA_2_3L19, respectively.

Mitogenome assembly was performed using a subsample of the whole genome sequencing using mode *all* of MitoZ (Meng et al. 2019). Despite various attempts, it was not possible to obtain a single contig from the NGS data alone, with a final assembly being constituted of two contigs. To scaffold over the two contigs, two primers matching the edge of both contigs, P8F 5′-GGTATTTGGTGCTTGAGC-3′ P8R and 5′-GCTAACACAAATACCCAGTCCG-3′, were designed and used for PCR amplification and Sanger sequencing. Each PCR reaction contained 2.5 μL 10× Invitrogen PCR Buffer, 1.5 μL 50 mmolL^−1^ MgCl_2_, 0.5 μL 10 mmolL^−1^ of each primer, and 10 mmolL^−1^ dNTPs, 0.1 μL Invitrogen Taq DNA Polymerase and approximately 1 μL DNA template. The cycle conditions (repeated for 36×) were as follows, initial denaturation at 94 °C for 3 min, denaturation at 94 °C (30 s), annealing at 54 °C (40 s), and extension at 72 °C (60 s) with a final extension at 72 °C for 10 min. Chromatograms were visually inspected using ChromasPro version 1.41 (technelysium.com.au) and scaffolding performed manually using BioEdit version 5.0.9 (Hall 1999). The final mitogenome was annotated in MITOS2 (Bernt et al. 2013) and manually validated by comparison with annotations from other Stomiiformes available at GenBank.

For phylogenetic analysis, all Stomiiformes mitogenomes (*n* = 7) and five outgroup taxa, were retrieved from GenBank (accessed in May 2020). BI and ML phylogenetic analysis were performed using all 13 protein-coding genes (PCG), individually aligned using GUIDANCE2 (Sela et al. 2015) with MAFFT (Katoh and Standley 2013) and concatenated in SequenceMatrix (Vaidya et al. 2011). The final alignment was 11,349 nt long. PartitionFinder2 on XSEDE (Lanfear et al. 2016) was applied to determine the partition schemes and best molecular evolutionary models for those partitions and the retrieved information used for phylogenetic analyses. These analyses were conducted using MrBayes on XSEDE (Ronquist et al. 2012) (GTR + I+G, GTR + I+G, HKY + I+G, and HKY + G) with two independent runs (10^7^ generations, sampling frequency one tree for 1000 generations) and RAxML-HPC BlackBox (Stamatakis, 2014) (GTR + I+G). Both phylogenetic analyses and PartitionFinder2 were implemented through CIPRES (Miller et al. 2010).

The mitogenome of *S. diaphana* here obtained represents the first mitogenome of a deep-sea fish from the family Sternoptychidae and has been deposited in GenBank (MT588184).

The mitogenome length (control region excluded) is 17,224 bp, within the observed size of other Stomiiformes mitogenomes (Miya et al. 2001; Miya and Nishida 1999, 2000; Aguilar et al. 2018; Ijichi et al. 2018). Regarding gene content, as expected 13 PCGs and 2 ribosomal RNA genes are present. Although 22 transfer RNAs were also annotated, we could not detect tRNA-Phe and two copies of tRNA-Met were differently positioned in the mitogenome. Furthermore, the *S. diaphana* mitogenome shows a completely new gene rearrangement among vertebrates (Satoh et al. 2016): -*trnC -trnQ nad2 -trnY trnW cox1 trnD -trnS2 cox2 trnK atp8 atp6 cox3 trnG nad3 trnR nad4l nad4 trnH trnS1 trnL1 nad5 -trnE cob trnT nad1 trnI trnM trnL2 trnN trnA -nad6 -trnP -trnM rrnS trnV rrnL*. Notwithstanding, despite several attempts, we could not circularize the mitogenome and the control region could not be detected. The inability to detect both the control region and the tRNA-Phe (generally adjacent to the control region) may result from the limitation of using Illumina short-read sequencing (Goodwin et al. 2016) and therefore requires future validation.

Both BI and ML phylogenetic trees, rooted with *Coregonus lavaretus* (Linnaeus, 1758), Salmonidae (following Ijichi et al. 2018), show the same topology (Figure 1). The phylogenetic analysis separates with high support, the group Stomiiformes from a cluster formed by families Synodontidae, Ateleopodidae, Myctophidae, and Trachipteridae.

**Figure 1.**
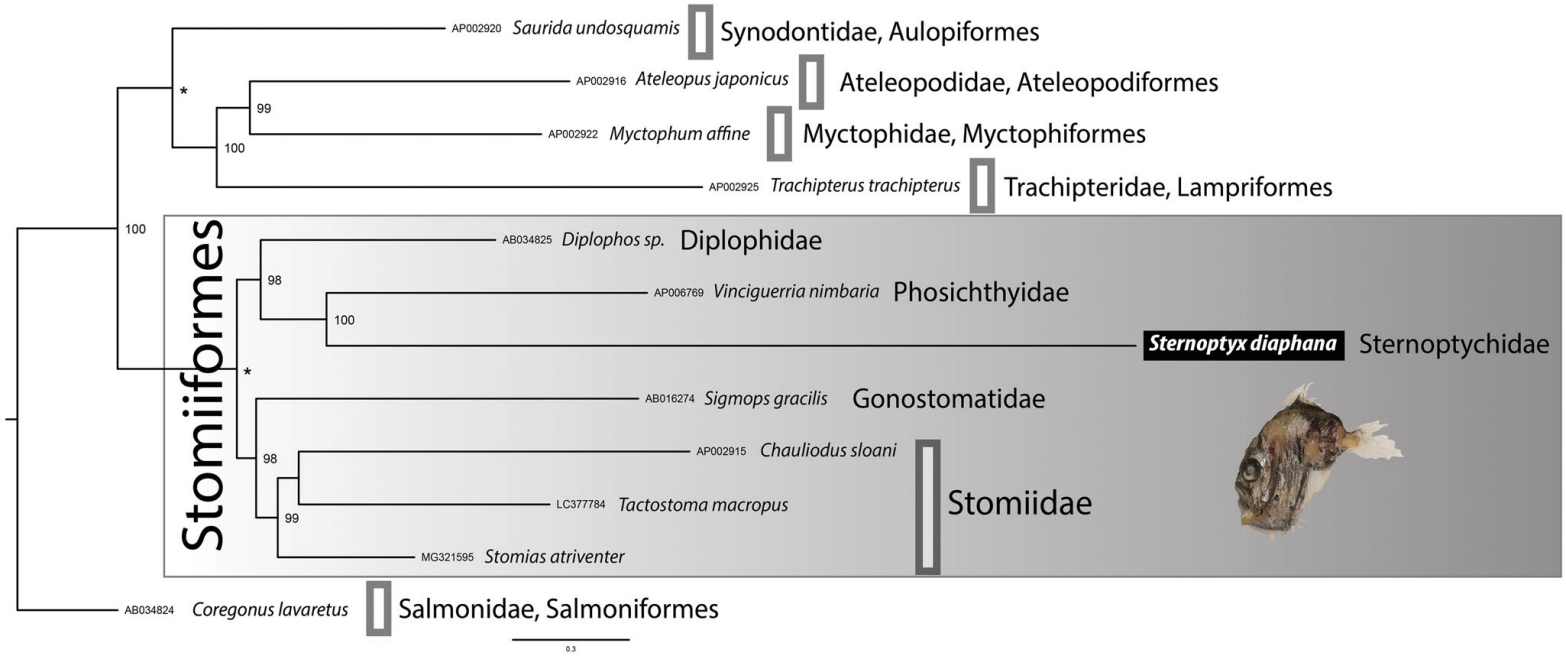
Bayesian Inference phylogenetic tree based on 13 concatenated protein-coding genes from 12 mitogenomes, including 7 Stomiiformes species and five outgroup taxa. Only supports above 92% are shown. The asterisk (*) on the nodes indicate that both posterior probabilities and bootstrap support values are 100%.

The Order Stomiiformes is divided into two well-supported groups, one comprising two families, i.e. Gonostomatidae and Stomiidae, and the other including Phosichthyidae, Diplophidae, and Sternoptychidae, represented by the here newly sequenced species *S. diaphana*. Although we have included all Stomiiformes available mitogenomes, this number is still reduced reinforcing the need to increase data sampling.

## Data Availability

The data that supports the findings of this study are available in GenBank of NCBI at https://www.ncbi.nlm.nih.gov, reference number MT588184 or from the corresponding authors, Elsa Froufe and L. Filipe C. Castro.
